# One-pot synthesis of Bi-Ni nanowire and nanocable arrays by coelectrodeposition approach

**DOI:** 10.1186/1556-276X-7-130

**Published:** 2012-02-15

**Authors:** Yuying Jia, Dachi Yang, Bin Luo, Shaomin Liu, Moses O Tade, Linjie Zhi

**Affiliations:** 1 National Center for Nanoscience and Technology, No.11, Beiyitiao Zhongguancun, Beijing, 100190, People's Republic of China; 2 Department of Chemical Engineering, Curtin University, Perth, Western Australia, 6102, Australia

**Keywords:** Bi-Ni nanocables, Bi-Ni nanowires, AAO, coelectrodeposition

## Abstract

A novel and convenient one-pot electrodeposition approach has been developed for precisely controlled fabrication of large-scale Bi-Ni nanowire and nanocable arrays. Using porous anodic aluminum oxide as a shape-directing template, by simply changing the electrochemical deposition mode, desired Bi-Ni hybrid nanowires and Bi-Ni core-shell nanocables have been obtained in the CV and CC modes, respectively. The structure, morphology, and composition of the as-prepared samples were characterized using X-ray powder diffraction, transmission electron microscopy, elemental mapping, and energy-dispersive X-ray spectrometry.

## Introduction

One-dimensional hetero-nanostructures, such as nanocables [1-3], nanowires [4-6], superlattice nanowires [7-9], and nanobelts [10-13], have attracted great interest in recent years. Among various fabrication strategies, electrochemical template deposition is a simple and versatile method because the well-defined templates allow us to control the length, diameter, and component of one-dimensional [1D] nanostructures. Recently, Xue et al. [14] developed a pulsed electrodeposition technique for the synthesis of Bi/Sb superlattice nanowire arrays with longitudinally ordered heterostructures. They synthesized four kinds of a modulated structure of Bi/Sb superlattice nanocables with different periods. Almost at the same time, Wang et al. [15] demonstrated the synthesis of Cu/Ni nanocables by codepositing nickel and copper atoms into the pores of anodic alumina membranes. Other heterostructures, such as Pd/Fe [16], Pd/Ni [17], ZnO/Cu_2_O [18], CdS/SnS [19], and CdS/TiO_2 _[20], have also been prepared by electrodeposition. However, since a depositing metal is different from a depositing semiconductor, it is still a critical challenge to controllably produce metal/semiconductor nanostructures with a designed morphology in one-step electrodeposition.

Bismuth (Bi) is a semimetal with unusual electronic properties that result from its highly anisotropic Fermi surface, low carrier concentration, small effective mass, and long mean free path of the carriers [21]. Because of these unique features, Bi has been extensively investigated for quantum transport, finite-size effects, and giant magnetoresistance effects [22]. Furthermore, Lee et al. [23] observed a semimetal-to-semiconductor transition when the diameter of Bi nanowire was smaller than 63 nm. The Bi-Ni heterojunction formation is therefore highly attractive not only for the incorporation of a magnetic property [24], but also for the introduction of semimetal and semiconductive behaviors into the structures. However, due to the relatively low conductivity of semimetal and semiconductive species, the one-step preparation of a metal/semiconductor heterojunction is challenging, particularly for the controllable formation of 1D nanocables. In this paper, we report a facile one-pot electrodeposition and anodic aluminum oxide [AAO] template-assisted method for growing uniform and well-aligned 1D Bi-Ni heterojunctions at a low temperature. 1D Bi-Ni hybrid nanowires and Bi-Ni core-shell nanocables have been successfully fabricated in a controlled fashion just by simply changing the electrodeposition conditions.

## Experimental details

### Fabrication process of the AAO template

All reagent are of analytical grade and used without further purification. AAO template was prepared by a two-step anodization method. A high-purity Al foil (99.999%, Beijing Mengtai Technology Development Co., Ltd., Beijing, China) was first annealed at 500°C for 4 h under nitrogen atmosphere followed by electropolishing with a voltage of 5 V in a mixture of HClO_4 _and C_2_H_5_OH (1:3 *v*/*v*). The electropolished Al foils were subjected to the first-step anodization for 4 h in a solution of 0.3 M oxalic acid (40 V, 0°C, graphite as a cathode). The first-step anodized layer was removed by etching in a mixture of phosphoric acid and chromic acid at 60°C for 8 to 10 h. The samples were thoroughly rinsed in distilled water and anodized again in 0.3 M oxalic acid (40 V, 0°C, graphite as a cathode). After the second-step anodization, the unwanted aluminum matrix was dissolved in a saturated CuCl_2 _solution at room temperature. Finally, the template was rinsed with distilled water and immersed in 5% phosphoric acid for about 20 to 40 min at 65°C to adjust the pore diameter and remove the barrier layer at the bottom of nanoholes.

### One-step synthesis of Bi-Ni nanowire and nanocable arrays

**Figure 1:**
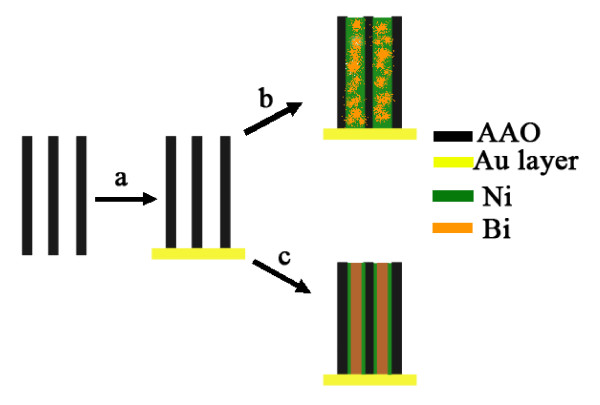
**Schematic for the synthesis of nanowires (top) and nanocables (bottom) via nanochannel-confined electrodeposition**. (**a**) Sputtering an Au layer on the bottom side of an AAO template. Electrodeposition process under (**b**) CV and (**c**) CC modes.

A CHI 660C electrochemical workstation with a standard three-electrode system (Chenhua Instruments, Shanghai, China) was used for the one-step electrodeposition. A saturated calomel electrode in a saturated KCl solution and a platinum foil were used as the reference electrode and the counter electrode, respectively. Figure 1 shows a schematic diagram of the synthesis processes. A thin layer of Au was deposited by evaporation onto one side of the through-hole AAO template to serve as the working electrode. Bi-Ni nanowire and nanocable arrays were electrodeposited into the AAO template from a solution containing 50 g/L NiSO_4_·6H_2_O, 20 g/L H_3_BO_3_, 37.5 g/L Bi(NO_3_)_3_·5H_2_O, 125 g/L C_3_H_5_(OH)_3_, 50 g/L C_4_H_6_O_6_, and 65 g/L KOH under constant voltage [CV] and constant current [CC] modes, respectively.

### Characterization

The morphology of the oriented Bi-Ni nanowire and nanocable arrays was characterized using an S-4800 field-emission scanning electron microscope [FE-SEM] (Hitachi, Chiyoda-ku, Tokyo, Japan). Prior to SEM observation, several drops of a 1-M NaOH aqueous solution were added onto the sample to dissolve the AAO template. The residual solution on the surface of the template was rinsed with distilled water. The structure of the resulting material was characterized by X-ray diffraction [XRD] (Rigaku, Shibuya-ku, Tokyo, Japan) with Cu Kα radiation at room temperature. Further detailed structural information of the oriented Bi-Ni nanowire and nanocable arrays was obtained using a Tecnai G2 F20 U-TWIN field-emission transmission electron microscope [TEM] (FEI, Shanghai, China) equipped with an energy-dispersive X-ray spectroscopy [EDS] system, digital scanning attachment, and high-angle annular dark field [HAADF] detector. In this paper, we have focused on the structural and compositional analyses of the nanowire and nanocable distribution using a combination of EDS in a scanning electron transmission microscopy [STEM] mode. For TEM observation, the template was completely dissolved in a 2-M NaOH aqueous solution. The product was centrifuged, thoroughly washed with distilled water to remove residual NaOH, and then dispersed in alcohol with the aid of ultrasonic agitation for 30 min. After that, a drop of solution was dropped onto a copper grid covered by porous carbon.

## Results and discussion

**Figure 2:**
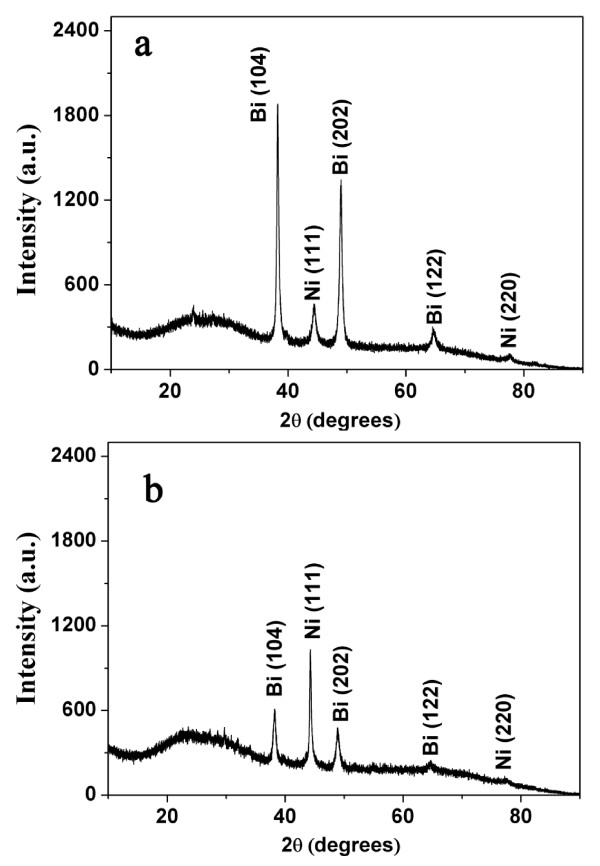
**A representative XRD pattern of the as-prepared Bi-Ni nanostructures, nanowire (a) and nanocable (b)**.

Typical XRD patterns of the as-prepared Bi-Ni nanostructure product embedded in AAO pores are shown in Figure 2. All diffraction peaks can be indexed to rhombohedral-structured Bi and cubic-structured Ni, which match very well with the reported values for Bi and Ni crystals (PDF card, Bi 44-1246, Ni 04-0850). No signals of impurities or side products were detected in the product. Influenced by the diffraction of the amorphous AAO template, an increasing background at 2*θ *between 20° and 30° can be observed in the XRD pattern [25]. Furthermore, the relative intensities of the peaks of nanowires are completely different from those of nanocables, implying that the different electrodeposition modes (CV or CC) have their own preferential crystal plane alignment when Bi and Ni nanostructures grow in the porous templates. It can be deduced that the reason may be that the growth orientation between the process of CV electrodeposition and that of CC electrodeposition is different [26].

**Figure 3:**
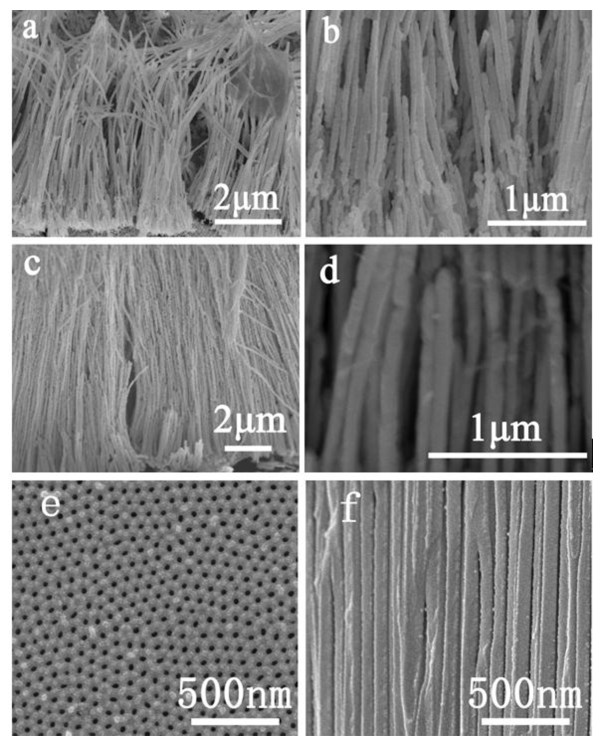
**SEM images of the as-prepared Bi-Ni nanostructure arrays and AAO template**. Side views of (**a**, **b**) nanowires and (**c**, **d**) nanocables. (b) and (d) are the magnified images of (a) and (c), respectively. (**e**) and (**f**) are the front and cross section of the AAO template, respectively.

Figure 3 shows typical SEM images of the Bi-Ni nanowire and nanocable products after the AAO template was removed. Large-area, continuous, smooth, highly ordered, and dense nanowire and nanocable arrays can be seen in Figure 3a, c, respectively. These arrays are all straight and parallel to each other and oriented vertically to the surface of the AAO template. The magnified images in Figure 3b, d show the morphologies of the nanowire/nanocables in a more distinguishable way. The size distribution of the as-prepared nanowires and nanocables are all uniform over the entire area observed, and the average diameter of the nanowires is about 50 nm in excellent consistency with the pore size dimension provided by the AAO template (as shown in Figure 3e, f).

**Figure 4:**
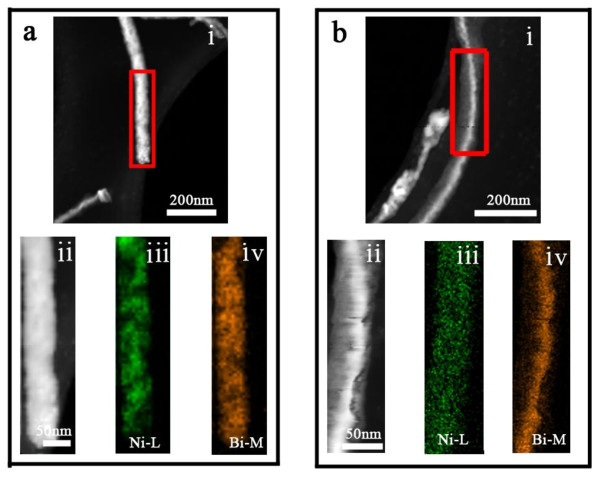
**Images of a Bi-Ni nanowire (a) and nanocable (b) obtained using STEM**. STEM HAADF images of (i) a Bi-Ni nanowire and nanocable and (ii) after an EDS image scan of the marked area in (i). Elemental mapping of the (iii) nickel and (iv) bismuth X-ray intensities compositing along the nanowire and nanocable.

Elemental mapping is one of the principal advantages of X-ray analysis as it provides clear and direct information regarding the distribution and quantity of each element in the sample. EDS image scanning across the whole nanostructure in the STEM mode was carried out to analyze the distribution of the chemical composition. Figure 4 shows STEM images and elemental maps of the Bi-Ni nanowire (Figure 4a) and nanocable (Figure 4b) specimens pasted on a holey carbon film. It displays a uniform distribution of bismuth and nickel in the whole wire range (Figure 4aiii, aiv). Figure 4ai exhibits the corresponding STEM HAADF image of the nanowire. The acquisition of an EDS image scan was approximately 36 min, and the drift was corrected by the FEI acquisition software. Hence, all HAADF images after an EDS scan showed a noticeable beam damage (Figure 4ii) and also some sample drift.

Figure 4b depicts the STEM images and elemental maps of the Bi-Ni nanocable specimen. As can be seen, nickel distribution is very uniform over the entire cable area (Figure 4biii); in contrast, bismuth tends to populate in the center of the cables (Figure 4biv). Figure 4bi gives the corresponding STEM HAADF image of the nanocable. Again, a minor amount of beam damage (Figure 4bii) and a sample drifting phenomenon were detected after the EDS scanning of these HAADF images.

**Figure 5:**
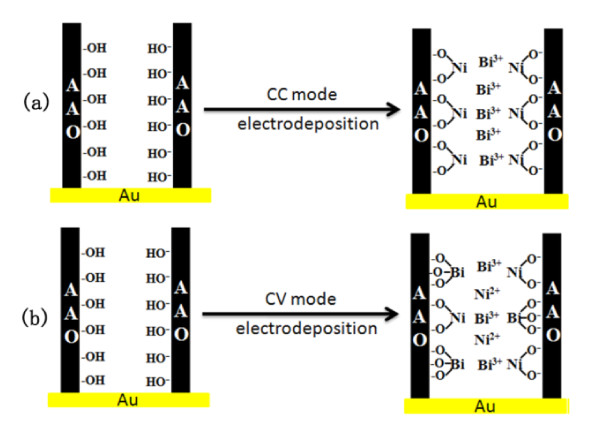
**The mechanism of forming Bi-Ni nanostructures under CC mode (a) and CV mode (b)**.

To explain the preferential growth of Bi and Ni in the nanopores of an alumina template, we propose that the preferential deposition of nickel on the pore walls could be a consequence of a chemical complexation of the Ni metal ions through hydroxyl groups on the pore wall [27,28], as shown in Figure 5a. Since the coupling constant of Bi^3+^/OH^- ^(15.8) is almost two times bigger than that of Ni^2+^/OH^- ^(8.55), Bi^3+ ^ions are thermodynamically more stable than Ni^2+ ^ions in a base solution. Therefore, in a CC mode, the Ni^2+ ^ions are push aside by Bi^3+ ^ions to the inner surface of AAO pores and deposited preferentially on the wall. This approach enables us to fabricate Bi-Ni core-shell nanocables by only one-step electrodeposition, and the ratio of Ni and Bi components in the nanocables can be easily controlled by varying the electrodeposition current density. The wall thickness and wall surface morphology of the nanotubes is also controllable since it simply depends on the Ni fraction. However, in the CV mode, both Ni^2+ ^and Bi^3+ ^were adsorbed on the pore wall due to the constant overpotential [29] (shown in Figure 5b). As a result, Bi-Ni hybrid nanowires were obtained.

## Conclusions

In summary, a novel and convenient one-pot electrodeposition approach has been developed for precisely controlled fabrication of large-scale Bi-Ni hybrid nanowire and Bi-Ni core-shell nanocable arrays. During the reaction process, AAO is used as a shape-directing template. By simply changing the electrochemical deposition mode, desired Bi-Ni hybrid nanowires and Bi-Ni core-shell nanocables have been obtained in the CV and CC modes, respectively. The present synthesis strategy may be extended to controllable fabrication of other 1D metal/semiconductor heterojunctions and their arrays.

## Abbreviations

AAO: anodic aluminum oxide; CC: constant current; CV: constant voltage; EDS: energy-dispersive X-ray spectroscopy; FE-SEM: field-emission scanning electron microscope; HAADF: high-angle annular dark field; STEM: scanning electron transmission microscopy; TEM: transmission electron microscope; XRD: X-ray diffraction.

## Competing interests

The authors declare that they have no competing interests.

## Authors' contributions

YJ carried out the nanowire and nanocable array experiment and prepared the manuscript. DY fabricated the anodic aluminum oxide membrane and participated in drafting the manuscript. BL provided the ideas on fabricated nanowire and nanocable arrays. SL provided the ideas on XRD analysis and participated in drafting the manuscript. MOT participated in drafting the manuscript. LZ conceived the study, participated in the experimental design and coordination, and helped draft the manuscript. All authors read and approved the final manuscript.
